# Mutated* WT1*,* FLT3-ITD,* and* NUP98-NSD1* Fusion in Various Combinations Define a Poor Prognostic Group in Pediatric Acute Myeloid Leukemia

**DOI:** 10.1155/2019/1609128

**Published:** 2019-07-30

**Authors:** Naghmeh Niktoreh, Christiane Walter, Martin Zimmermann, Christine von Neuhoff, Nils von Neuhoff, Mareike Rasche, Katharina Waack, Ursula Creutzig, Helmut Hanenberg, Dirk Reinhardt

**Affiliations:** ^1^Department of Pediatrics III, University Children's Hospital Essen, University of Duisburg-Essen, 45122 Essen, Germany; ^2^Department of Pediatric Hematology and Oncology, Hannover Medical School, 30625 Hannover, Germany; ^3^Department of Child and Adolescent Psychiatry, Psychosomatics and Psychotherapy, LVR Klinikum Essen, University Hospital Essen, University of Duisburg-Essen, Germany; ^4^Centre for Research Acceleration in Pediatrics GmbH, Hannover, Germany; ^5^Department of Otorhinolaryngology & Head/Neck Surgery, Heinrich Heine University, 40225 Duesseldorf, Germany

## Abstract

Acute myeloid leukemia is a life-threatening malignancy in children and adolescents treated predominantly by risk-adapted intensive chemotherapy that is partly supported by allogeneic stem cell transplantation. Mutations in the* WT1* gene and* NUP98-NSD1* fusion are predictors of poor survival outcome/prognosis that frequently occur in combination with internal tandem duplications of the juxta-membrane domain of* FLT3* (*FLT3-ITD). *To re-evaluate the effect of these factors in contemporary protocols, 353 patients (<18 years) treated in Germany with AML-BFM treatment protocols between 2004 and 2017 were included. Presence of mutated* WT1* and* FLT3-ITD *in blasts (n=19) resulted in low 3-year event-free survival of 29% and overall survival of 33% compared to rates of 45-63% and 67-87% in patients with only one (only* FLT3-ITD; n=33, *only* WT1* mutation; n=29) or none of these mutations (n=272). Including* NUP98-NSD1* and high allelic ratio (AR) of* FLT3-ITD* (AR ≥0.4) in the analysis revealed very poor outcomes for patients with co-occurrence of all three factors or any of double combinations. All these patients (n=15) experienced events and the probability of overall survival was low (27%). We conclude that co-occurrence of* WT1* mutation,* NUP98-NSD1,* and* FLT3-ITD *with an AR ≥0.4 as triple or double mutations still predicts dismal response to contemporary first- and second-line treatment for pediatric acute myeloid leukemia.

## 1. Introduction

Pediatric acute myeloid leukemia (AML) is a rare and heterogeneous disorder, for which continuous improvement of risk-adapted treatment approaches over the last 30 years has led to overall survival rates of approximately 70% [1, 2]. In current pediatric AML treatment protocols, cytogenetic abnormalities of the leukemic blasts at initial diagnosis are important indicators for risk group stratification and treatment assignment [1, 2]. Approximately, 25% of pediatric patients have AML blasts with a normal karyotype, but even these cases often harbor somatic mutations in genes such as* WILMS TUMOR 1 *(*WT1*),* NPM1*,* NRAS, KRAS*,* Fms-like tyrosine kinase 3* (*FLT3),* and/or* c-KIT/CD117 *[1, 2].

The* WT1* gene is located on chromosome 11, has ten exons and four zinc finger domains, and functions as a transcription factor and master regulator of tissue development [3]. Within normal hematopoiesis,* WT1* has two distinct roles: in early stages, it mediates quiescence of primitive progenitor cells, and later, WT1 expression is important for differentiation towards the myeloid lineage [4]. In AML,* WT1* mutations are present in approximately 10% of patients and predominantly located in exons 7 and 9, which contain the DNA-binding zinc finger domains of the protein. The majority of these mutations are out-of-frame deletion/insertions or premature termination codons that will lead to truncated proteins with altered functional consequences for the cells [5]. If these truncated proteins are stable, they might have dominant negative effects by partially blocking the wild-type WT1 protein; if unstable, the diminished WT1 protein levels may lead to haploinsufficiency [5]. Nevertheless, it has been clearly established that the occurrence of* WT1* mutations in AML blasts with normal karyotypes is associated with adverse clinical outcomes in adult [6–9] as well as pediatric patients [10, 11].

Somatic* WT1 *mutations in AML blasts often co-occur with other genetic aberrations, most frequently with an internal tandem duplication in the juxta-membrane domain of the tyrosine kinase receptor* FLT3* (*FLT3-ITD*) [5]. Classified as type-I or proliferating mutation,* FLT3-ITDs *are present in 10-15% of pediatric AML cases and lead to poor clinical outcomes [12–14]. We previously demonstrated in a cohort of 298 pediatric patients with* de novo *AML treated before 2004 on AML-BFM protocols that the combination of* FLT3-ITD* and mutated* WT1* is associated with even worse survival [10]. Comparably, an independent study from the Children's Oncology Group (COG) in a cohort of 842 children with* de novo *AML showed that the poor prognostic impact of* WT1 *mutations depends on the* FLT3-ITD* status [11]. These two pediatric studies confirmed earlier findings in adults that first established the adverse prognostic impact of both* WT1 *and* FLT3-ITD *mutations [15, 16].

Two additional prognostic indicators in* FLT3-ITD-*positive AML cases established in the last few years are the mutational burden in each patient defined as the ratio between mutant and wild-type* FLT3-ITD* alleles (allelic ratio, AR) [12, 17, 18] and the co-occurrence of* FLT3-ITD* with a cytogenetically cryptic translocation of chromosomes 5 and 11 or t(5;11)(q35;p15) [19]. This translocation leads to fusion of the* nucleoporin* (*NUP98*) gene on chromosome 11 and the gene for nuclear receptor binding SET-domain protein 1 (*NSD1*) of chromosome 5 (*NUP98-NSD1*). As the breakpoints for the NUP98 gene are often not detected by classical cytogenetic due to its terminal localization at 11p15, it has been described in AML cases with a “normal” karyotype [20]. Importantly, this rare recurrent aberration is mutually exclusive with other recurrent translocations and more prevalent in pediatric AML, in which it is associated with the presence of* FLT3-ITD* and poor survival outcomes [21, 22].

In the present study, we re-evaluated the role of mutations in* WT1*,* FLT3-ITD,* and the* NUP98-NSD1* translocation as prognostic factors in two contemporary pediatric treatment protocols by analyzing their association with co-occurring genetic and cytogenetic aberrations and by determining their clinical significance and influence on treatment outcome. Thereby, we were able to define a group of high-risk patients for which the efforts for salvage/second line treatment largely failed.

## 2. Materials and Methods

**Figure 1:**
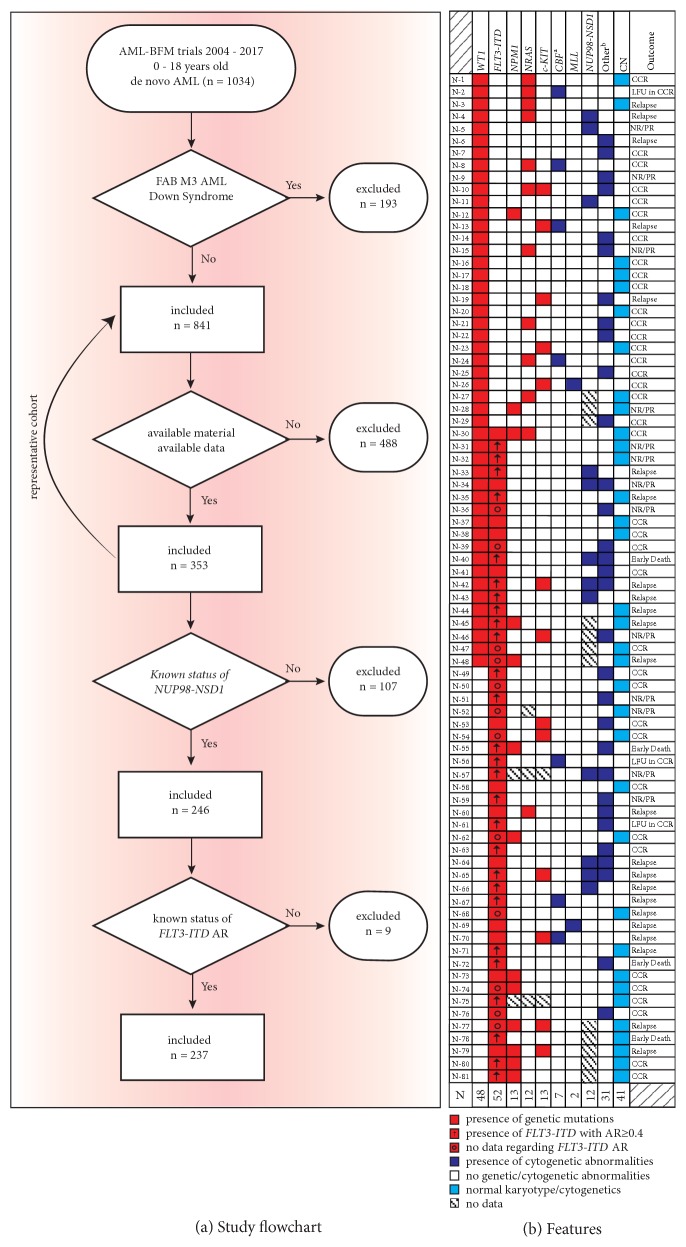
*Study flowchart and patient characteristics.* (a) Study flowchart outlining the process of patient recruitment in the data analysis. (b)* WT1* mutations often co-occurred with* FLT3-ITD* and other genetic aberrations. AML-BFM, acute myeloid leukemia-Berlin-Frankfurt-Muenster; n, number;* WT1*,* Wilms Tumor 1*;* FLT3-ITD*, fms-related tyrosine kinase 3-internal tandem duplication;* NPM1*,* nucleophosmin 1*;* NRAS*,* neuroblastoma RAS viral oncogene homolog*;* c-KIT*,* KIT proto-oncogene*; CBF, core binding factor; MLL, rearrangements of* MLL* gene;* NUP98-NSD1*,* Nucleoporin-Nuclear Receptor Binding SET Domain Protein 1 *fusion gene; CN, cytogenetic-normal AML; AR, allelic ratio; CCR, continued complete remission; LFU, lost to followup; NR, non-response; PR, partial remission. ^a^CBF aberrations include translocation of chromosomes 8 and 21 and inversion or translocation of chromosome 16. ^b^Other cytogenetic aberrations such as trisomy 8, various chromosomal translocations, and complex karyotype alterations.

From April 2004 to May 2017, 841 patients aged 0–18 years with* de novo* AML (excluding FAB M3 and Down Syndrome) were treated in Germany according to the AML-BFM 04 trial (ClinicalTrials.gov Identifier: NCT00111345) or the AML-BFM 2012 registry and trial (EudraCT number: 2013-000018-39) (Figure 1(a)). Both trials were approved by the ethical committees and institutional review boards of university hospitals of Münster and Hannover and an informed consent was obtained from each patient or their legal guardians before the beginning of treatment. Standard procedures for the diagnosis of AML were carried out by the German AML-BFM reference laboratory as previously described [23–25]. This included mutation analysis in* WT1*,* FLT3-ITD*,* NPM1*,* NRAS,* and* c-KIT* by Sanger and/or next-generation sequencing or GeneScan analysis. In 353 patients (42%), sufficient material and clinical data were available for further analysis. As a confirmation, material from* WT1* and/or* FLT3-ITD *positive and negative cases was re-analyzed by next-generation sequencing (NGS) using the TruSight Myeloid Panel (Illumina)[26] with median read counts for* WT1 *and* FLT3-ITD* of around 4,200 and 6,000 reads, respectively, as we described previously [27]. In addition, the allelic ratio of* FLT3-ITD *to* FLT3 *wild-type was calculated via GeneScan analysis [13] and the expression of* NUP98-NSD1* was analyzed in 246 out of 353 patients with available material by real-time quantitative PCR using previously described primers [19]. Initial analysis demonstrated that the selected cohort was representative for all patients treated between 2004 and 2017 on the AML-BFM protocols for features such as gender, age, AML subtype, initial cytogenetics, and preliminary, early response to treatment (data not shown).

Clinical end-points were defined as previously described [28, 29] and survival rates were calculated via Kaplan-Meier analysis and compared by log-rank test. Multivariate analysis was performed using Cox regression model evaluating the hazard ratio (HR) of each covariate with 95% confidence interval (CI). Stem cell transplantation was included in the Cox regression model as a time-dependent variable. Differences with a p value less than 0.05 were considered as significant. Data were analyzed using the Statistical Analysis System software version 9.4 (SAS Institute, Cary, NC). Data acquisition was stopped at June 30, 2018, with a median follow-up of 3.6 years.

## 3. Results

### 3.1. Study Cohort and Patient Characteristics

In this study, we included 353 patients treated on either the AML-BFM 2004 or AML-BFM 2012 protocol for whom sufficient material and information were available (Figure 1(a)). As shown in Table 1, 48 (14%) patients had* WT1 *and 52 (15%)* FLT3-ITD *mutations in their leukemic blasts at diagnosis. Mutations in* NPM1*,* NRAS,* and* c-KIT* were present in the blasts of 9%, 17%, and 12% of patients, respectively. Most patients with mutated* WT1* (n=35, 73%) harbored at least one co-occurring mutation in the AML blasts, with the most common being* FLT3-ITD* (n=19, 40%) followed by* NRAS* mutations (n=11, 23%, Table 1 and Figure 1(b)). Comparably, the majority of patients with* FLT3-ITD* had additional mutations in other genes (n=32, 62%), most commonly in* WT1* (n=19, 37%) and* NPM1* (n=11, 21%). Patients with mutated* WT1 *or* FLT3-ITD* were older compared to the rest of the study cohort, and AML FAB M1/M2 was the most common morphologic subtype in both groups (Table 1). In addition, the AML blasts of more than half of patients with* WT1 *(n=25/48, 52%) and* FLT3-ITD *(n=28/52, 54%) mutations had a normal karyotype at diagnosis; these percentages were significantly higher than those in patients without mutations in each of the two genes (p<0.0001, Table 1).

### 3.2. Characteristics of WT1 Mutations

We identified 64 different* WT1* sequence alterations in 48 patients (Table 2). These alterations were frequently located in exon 7 (n=55, 86%) and predominantly resulted in frameshifts producing premature termination codons (PTCs). In total, nine single nucleotide variants (SNVs) were found, mostly in exon 9 (n=7, 78%). Only three of the nine SNVs were not previously reported as pathogenic (Table 2). Using NGS, we characterized multiple distinct* WT1* mutations with highly diverse variant allele frequencies in 13 patients (11 patients had two and 2 patients, three distinct mutations). We then analyzed the heterozygosity of these mutations via the integrative genomic viewer (Broad Institute, MA, USA) and determined that they were all located on individual/different alleles/reads (Table 2).

### 3.3. Survival Significance of the Genomic Aberrations

**Figure 2:**
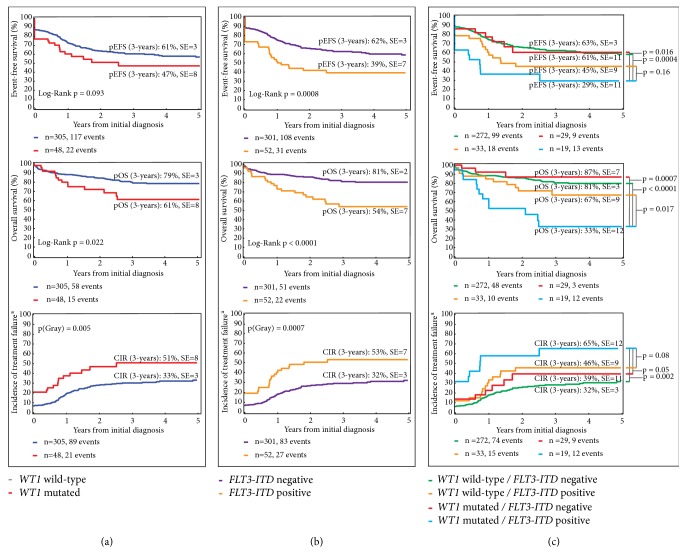
*Co-occurrence of WT1 and FLT3-ITD mutations at initial diagnosis of pediatric AML predicts poor survival outcomes. *(a)* WT1* mutation as single factor increased the incidence of relapse, reducing the probability of survival. (b) The presence of* FLT3-ITD*, individually, leads to an increased chance of relapse and decreased patient survival. (c) Clinical consequences of* WT1* mutations and* FLT3-ITD* were dependent on each other.* WT1*,* Wilms Tumor 1*;* FLT3-ITD*, fms related tyrosine kinase 3-internal tandem duplication; pEFS, probability of event-free survival; pOS, probability of overall survival; CIR, cumulative incidence of relapse; SE, standard error; n, number. ^a^No response to treatment was considered as the occurrence of an event at time zero.

**Figure 3:**
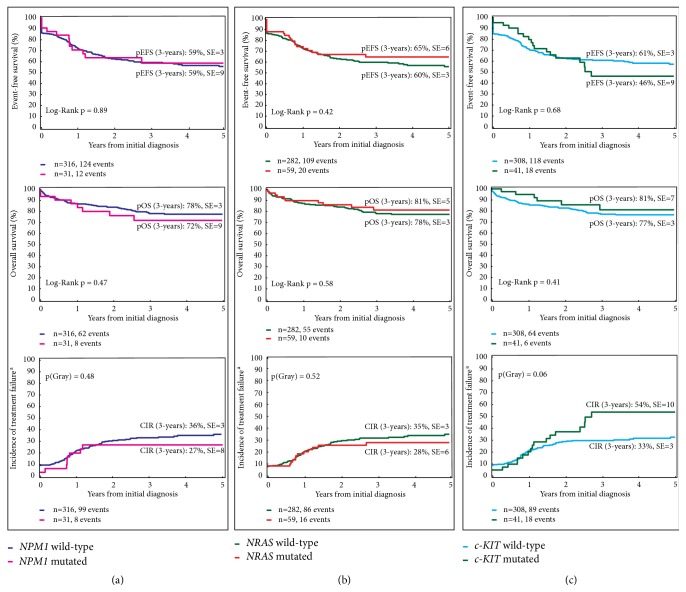
*Mutations in NPM1, NRAS, and c-KIT had no impact on survival. *(a) Prognostic impact of mutated* NPM1* on EFS, OS, and CIR. (b) Prognostic impact of mutated* NRAS* on EFS, OS, and CIR. (c) Prognostic impact of* c-KIT* mutation on EFS, OS, and CIR.* NPM1*,* nucleophosmin 1*;* NRAS*,* neuroblastoma RAS viral oncogene homolog*;* c-KIT*,* KIT* protooncogene; pEFS, probability of event-free survival; pOS, probability of overall survival; CIR, cumulative incidence of relapse; SE, standard error; n, number. ^a^No response to treatment was considered as the occurrence of an event at time zero.

Next, we analyzed the impact of each mutation on the clinical outcomes. Our analysis identified* WT1 *and* FLT3-ITD, *but not* NRAS*,* NPM1,* or* c-KIT* mutations as single factors that significantly increased the chance of relapse or treatment failure and reduced the probability of 3-year overall survival (OS) in our patient cohort (Figures 2(a), 2(b), and 3). In addition,* FLT3-ITD *but not* WT1 *mutations significantly decreased the 3-year probability of event-free survival (EFS, Figure 2(b)). When we grouped the two mutations together, the survival analysis revealed a 3-year EFS of 29±11% for patients with both* WT1 *and* FLT3-ITD *mutations compared to 63±3% for patients with none of these mutations (p=0.0004) and 61±11% or 45±9% for patients with only* WT1 *mutation (p=0.016) or* FLT3-ITD* (p=0.16), respectively (Figure 2(c)). Corresponding to this low EFS, co-occurrence of these two mutations was associated with an increased cumulative incidence of relapse (CIR) of 65±12% compared to 32±12% for patients with none of these mutations (p=0.002) and 39±11% or 46±9% for patients with only* WT1 *mutation (p=0.05) or* FLT3-ITD *(p=0.08), respectively (Figure 2(c)). Furthermore, we identified a low 3-year OS probability of 33±12% in patients with co-occurrence of* WT1 *and* FLT3-ITD*, which was significantly lower than those of patients without these mutations (81±3%, p<0.0001), patients with only mutated* WT1 *(87±7%, p=0.0007), and patients with only* FLT3-ITD *(67±9%, p=0.017, Figure 2(c)). Comparing the curves for EFS and OS clearly demonstrated that our second line treatment was not able to rescue any patient with co-occurrence of* WT1 *and* FLT3-ITD* mutations, while the OS rates increased by more than 20% for the other three subgroups (Figure 2(c)).

### 3.4. Impact of NUP98-NSD1 Fusion

To further characterize the prognostic significance of* WT1 *and* FLT3-ITD *mutations, we analyzed the expression of* NUP98-NSD1 *fusion in our patient cohort (Figure 1(a)). From 246 patients with available material for this retrospective real-time quantitative PCR analysis, 15 (6%) of them were identified to have the* NUP98-NSD1 *translocation. Most of these patients (12/15, 80%) harbored additional* WT1* or* FLT3-ITD *mutations: 3 patients carried both* WT1* and* NUP98-NSD1*, 4 had a co-occurrence of* FLT3-ITD *and* NUP98-NSD1*, and 5 patients carried all three genetic alterations (Figure 1(b)). Only 1 of these 15 patients had a previous known status of* NUP98-NSD1* by conventional karyotyping: 2 others were previously diagnosed with deletion of chromosome 5, 1 carried an inversion of chromosome 16 (no other mutations and still in continuous complete remission), 4 carried complex karyotypes or rare aberrations, and 7 had no other cytogenetic abnormalities (data not shown).

**Figure 4:**
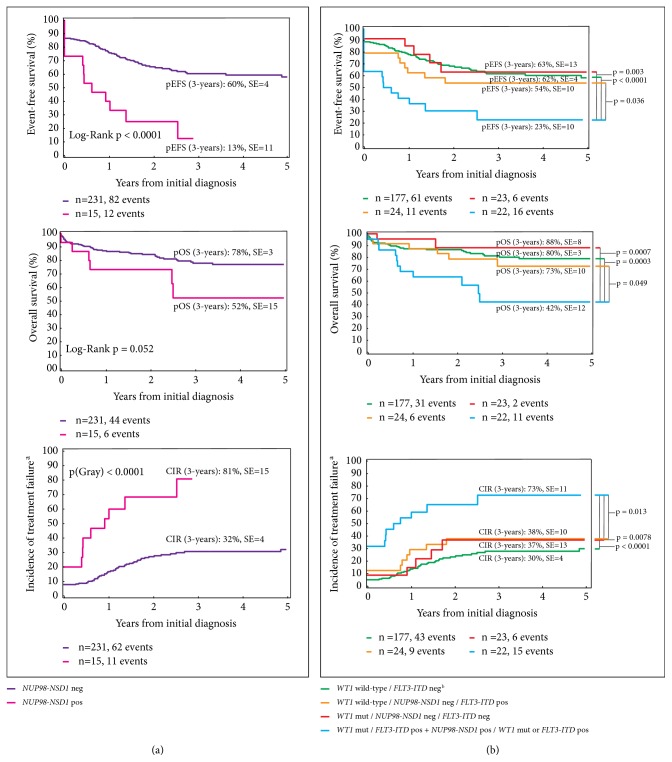
*Prognostic significance of NUP98-NSD1 fusion.* (a)* NUP98-NSD1* as single factor predicted poor outcomes. (b) Inclusion of* NUP98-NSD1* as poor prognostic factor with* WT1* mutation and* FLT3-ITD*, predicted poor outcomes for patients harboring all three factors in addition to patients with* NUP98-NSD1* and* WT1* mutation or* FLT3-ITD*. Patients with unknown status of* NUP98-NSD1* fusion were excluded from this analysis. W*T1*,* Wilms Tumor 1*;* FLT3-ITD*, fms related tyrosine kinase 3-internal tandem duplication;* NUP98-NSD1*,* Nucleoporin-Nuclear Receptor Binding SET Domain Protein 1* fusion gene; pEFS, probability of event-free survival; pOS, probability of overall survival; CIR, cumulative incidence of relapse; SE, standard error; mut, mutated; pos, positive; neg, negative. ^a^No response to treatment was considered as the occurrence of an event at time zero. ^b^Three patients with* NUP98-NSD1 *are included in this group.

We then analyzed the prognostic significance of* NUP98-NSD1* in the cohort of 246 patients with the known status of this fusion gene (Figure 1(a)). As a single factor, the presence of* NUP98-NSD1 *in AML blasts of patients at diagnosis was associated with a significant increase in CIR (81%) in addition to decreased probabilities of 3-year EFS and OS (Figure 4(a)). Combining* NUP98-NSD1 *with* WT1* and* FLT3-ITD *mutations in our multifactor survival analysis revealed that patients with all three or either two of these mutations had worse survival outcomes. These patients had a higher CIR of 73±11% compared to the CIR of 30±4% for patients with none of these aberrations or* NUP98-NSD1 *alone (p<0.0001) and the CIR of 37±13% or 38±10% for patients with only mutated* WT1 *(p=0.0078) or* FLT3-ITD *(p=0.013), respectively (Figures 4(a) and 4(b)). The increased CIR translated into a lower 3-year EFS probability of 23±10% for patients with triple or double mutations compared to the EFS of 62±4% for patients with none of these mutations or only* NUP98-NSD1* (p<0.0001) and the EFS of 63±13% or 54±10% for patients with only* WT1* (p=0.003) or* FLT3-ITD* (p=0.036) mutations, respectively (Figure 4(b)). Moreover, co-occurrence of all three or any double mutations resulted in a significantly lower 3-year OS probability of 42±12% compared to 80±8% for patients with none of the mutations or only* NUP98-NSD1 *(p=0.0003) and 88±8% or 73±10% for patients with only* WT1* (p=0.0007) or* FLT3-ITD *(p=0.049) mutations, respectively (Figure 4(b)).

### 3.5. Survival Significance of the FLT3-ITD Allelic Ratio

**Figure 5:**
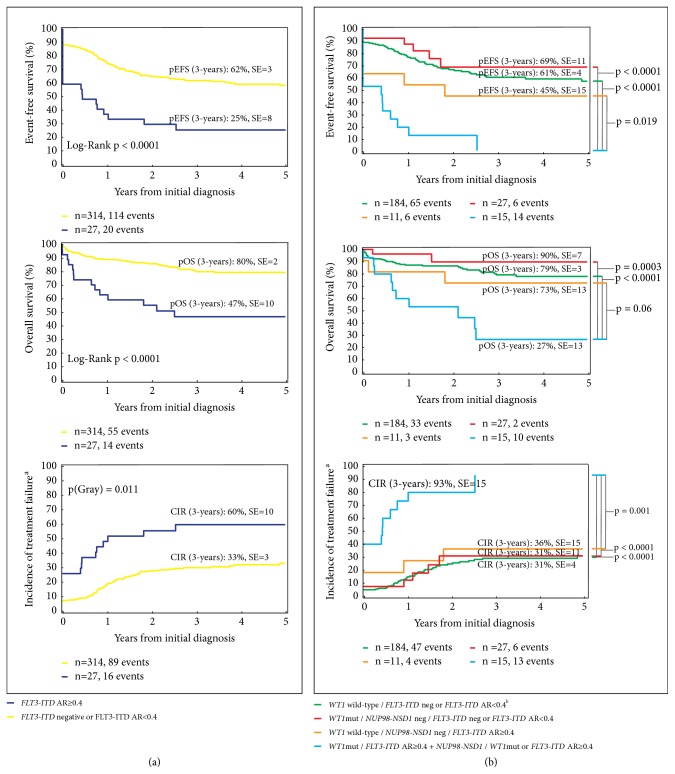
*Prognostic significance of mutational burden of FLT3-ITD.* (a)* FLT3-ITD* with an allelic ratio ≥0.4 as a single factor predicted poor outcomes. (b) High mutational burden of* FLT3-ITD* was another predictor of poor prognosis when it occurred with* WT1* and/or* NUP98-NSD1*. Patients with an unknown* FLT3-ITD* AR were excluded from this analysis.* NUP98-NSD1*,* Nucleoporin-Nuclear Receptor Binding SET Domain Protein 1* fusion gene;* FLT3-ITD*, fms related tyrosine kinase 3-internal tandem duplication; pEFS, probability of event-free survival; pOS, probability of overall survival; CIR, cumulative incidence of relapse; AR, allelic ratio; SE, standard error; n, number. ^a^No response to treatment was considered as the occurrence of an event at time zero. ^b^Three patients with* NUP98-NSD1* are included in this group.

We have previously established the prognostic significance of an* FLT3-ITD* allelic ratio of ≥0.4 in pediatric AML [12]. Therefore, to determine the impact of the mutational burden of* FLT3-ITD* on treatment outcomes in the present cohort, we calculated the* FLT3-ITD* AR in patients with available data/material. As indicated in Figure 1(b), 27 patients had an AR ≥0.4 at diagnosis. Analyzing the survival impact of the* FLT3-ITD* AR ≥0.4 revealed that as a single factor, it was associated with an EFS of only 25±8% and an OS of only 47±10%, respectively (Figure 5(a)). Remarkably, the co-occurrence of* FLT3-ITD *AR ≥0.4,* WT1,* and* NUP98-NSD1* as triple or double mutations significantly increased the CIR to 93±15% compared to the CIR of 31±4% for patients with no mutations or only* NUP98-NSD1 *or* FLT3-ITD *AR <0.4 (p<0.0001) and to the CIR of 31±11% or 36±15% in patients with only* WT1* (p<0.0001) or* FLT3-ITD *AR ≥0.4 (p=0.001) mutations, respectively (Figure 5(b)). The probability of 3-year EFS was zero in patients with double or triple* WT1*,* FLT3-ITD *AR ≥0.4, and* NUP98-NSD1* mutations as opposed to 61±4% in patients with no mutations or only* NUP98-NSD1 *or* FLT3-ITD *AR <0.4 (p<0.0001) and 69±11% or 45±15% for patients with only mutated* WT1* (p<0.0001) or* FLT3-ITD *AR ≥0.4 (p=0.019), respectively (Figure 5(b)). Finally, the co-occurrence of double or triple mutations resulted in a 3-year OS probability of 27±13%, which was significantly lower than the 3-year OS of 79±3% in patients with no mutations or only* NUP98-NSD1 *or* FLT3-ITD *AR <0.4 (p<0.0001) and 90±7% or 73±13% in patients with only* WT1 *(p=0.0003) or* FLT3-ITD *AR ≥0.4 (p=0.06) mutations, respectively (Figure 5(b)). By multivariate analysis including* WT1 *mutation,* FLT3-ITD *AR ≥0.4, core-binding factor aberrations, early bone marrow response to treatment, and stem cell transplantation as covariables, we confirmed that the interaction of these three factors, and not each of the aberrations individually, was a significant predictor of poor prognosis for EFS (p=0.008, HR: 3.88, 95% CI: 1.42 – 10.6) and OS (p=0.042, HR: 3.42, 95% CI: 1.04 – 11.21, Table 3). Importantly, none of the patients with triple mutations survived and the only patients who could be rescued harbored double* NUP98-NSD1* and* WT1* or* NUP98-NSD1* and* FLT3-ITD* mutations (Figure 1(b)), thus resulting in an OS of 27±13% (Figure 5(b)).

## 4. Discussion

Treatment of pediatric AML has significantly improved over the past three decades due to the development of intensified first-line treatments, efficient second-line therapies, and optimized supportive care [2, 30]. The success is, at least partly, achieved by more efficient risk group stratification using factors such as somatic mutations and cytogenetic aberrations of AML blasts at diagnosis as well as considering the primary response to treatment to optimize the allocation of patients to standard or enhanced treatment options [1]. In the present study, we analyzed the influence of three parameters, mutations in* WT1 *and* FLT3 *and the translocation of* NUP98-NSD1*, on the outcome of pediatric patients in the German AML-BFM 2004 and 2012 protocols. Although all three parameters have been established by us and others as important prognostic factors in both pediatric and adult patients [8–14, 20–22], their combined utility to identify high-risk patients likely to experience dismal treatment results has not yet been reported in a contemporary pediatric AML trial.

In a cohort of 237 patients treated within the AML-BFM 2004 and 2012 protocols and with sufficient material for re-analysis, we observed favorable outcomes for 3-year EFS of 61% and 69% and OS of 79% and 90% in patients without* WT1* mutations or* NUP98-NSD1 *fusion or with only one of these factors. Patients with leukemic blasts that were* FLT3-ITD* positive but negative for* WT1* and* NUP98-NSD1* mutations and that had an* FLT3-ITD* AR ≥0.4 still achieved an EFS of 45% and an OS of 73%. Surprisingly, our data therefore suggests that without* WT1* and* NUP98-NSD1 *mutations, the negative impact of* FLT3-ITD* even with an AR≥0.4 might not be as severe as previously published [12, 17]. However, all patients positive for at least two of the three risk factors and with an* FLT3-ITD* AR ≥0.4 had events within the first three years and only 27% could be rescued by our salvage therapies. These unfavorable results in our double or triple mutated group unequivocally demonstrate that our current first-line treatment strategies for these patients are still insufficient/inadequate and urgently need improvement.

Of the three risk factors, currently only the* FLT3-ITD* mutation can be specifically targeted with inhibitors [31]. Although the first generations of these drugs only achieved limited and often transient efficacy due to intrinsic and extrinsic adaptations in the AML blasts and/or the environment [31], combination therapies of newer tyrosine kinase inhibitors such as Quizartinib with standard chemotherapy seem to be relatively well tolerated and in initial studies have demonstrated survival improvement in relapsed or refractory AML patients [32–34]. Due to the important role of FLT3 pathway activation in AML, numerous combinations of FLT3 inhibitors with other drugs are currently being tested. Whether these results will also be helpful for the treatment of pediatric AML will need to be carefully determined in future studies, especially considering the clonal heterogeneity of* FLT3-ITD* and the additional survival burden that it causes by increasing drug resistance through clonal evolution or selection and further expansion of resistant AML clones [35, 36]. Nevertheless, it is tempting to speculate that the simple addition of a newer FLT3 inhibitor to our standard therapy might be a feasible, well-tolerated, and effective approach for all patients with blasts that are positive for the* FLT3-ITD* mutation, regardless of the status of alterations in* WT1* or* NUP98*.

The role of WT1 in patients with AML is still controversial [4]. Although* WT1* is overexpressed in the majority of leukemias and can be used as a marker for minimal residual disease and maybe even vaccination attempts, the prognostic and therapeutic relevance of high or absent WT1 expression levels is not unequivocally accepted [37–39]. In contrast, mutations in* WT1* are clearly identified as determinants of poor prognosis and, as we showed here, confer a dismal prognosis especially in combination with* FLT3-ITD* or* NUP98-NSD1* fusion. In the present study, we identified 64 monoallelic* WT1 *sequence alterations in exon 7 or exon 9 in the leukemic blasts of 48 patients. The majority of these alterations leads to frameshifts and/or premature terminations codons and thus shortened proteins. These mutant proteins can act in a dominant negative manner [40], which may contribute to a myeloid differentiation block present in AML blasts [41]. However, similar mutations have also been described in the context of Wilms tumors as gain-of-function mutations promoting proliferation [42]. Here, we show a favorable prognosis for patients with single* WT1 *mutations, with 26 out of 29 cases reaching continued complete remission (CCR) (Figure 1(b)). Therefore, based on a 3-year EFS of 69% and an OS of 90%, the development of new treatment approaches is not as urgently needed for these patients with* WT1* mutated blasts that do not harbor* FLT3-ITD* or* NUP98-NSD1* mutations.

Among the 31 different fusion gene partners of* NUP98* identified so far, the* NUP98-NSD1* t(5:11) translocation is the most frequent and present in 4-7% of patients in pediatric AML patients [20–22]. Importantly, the* NUP98* translocations that occur in AML all share the N-terminus of the protein and are thought to initially lead to epigenetic dysregulation of different leukemia-associated genes including* HOXA7*,* HOXA9,* and* HOXA10* in myeloid precursor cells [20]. Additional somatic mutations in other genes occur as secondary events and promote malignant transformation and uncontrolled cell growth [20]. As also shown in our patient data set, these secondary alterations often include activating mutations in* FLT3 (FLT3-ITD) *or truncating mutations* in WT1* [21]. Strikingly, only three patients in our study had a* NUP98-NSD1* translocation without mutations in* FLT3* or* WT1*; two of these patients achieved and remained in first CCR at the end of data acquisition. The third patient had no other genetic risk factors but a very high initial white blood cell count of almost 400,000 cells/*μ*l. Complete remission induction was delayed, and the patient relapsed a year later but was successfully treated by allogeneic stem cell transplantation with a follow-up of 10 years. Therefore, as also described previously [21], our patients with* NUP98*-rearranged blasts with* WT1* and/or* FLT3-ITD* mutations had a poor prognosis, especially in contrast to patients with only* WT1* and* FLT3-ITD* mutations, who could at least partially be rescued by allogeneic transplantation. However, due to the high risk of failure of the first-line treatment, stem cell transplantation already in first CCR seems to be an attractive option for cases of* NUP98*-rearranged AML [21, 22]. Nevertheless, it should be noted that even allogeneic stem cell transplantation is not always effective in improving the treatment outcome in patients with a high probability of treatment failure based on risk stratification. Thus, introducing novel treatment approaches such as the use of small inhibitors, e.g., venetoclax and isadanutlin [43] or cellular therapies with allogeneic NK-cells or engineered T-cells with chimeric antigen receptors (CARs) [44] targeting leukemic blasts harboring* NUP98* rearrangement or* WT1 *mutations should be taken into consideration in future clinical studies.

Recent analysis from a collaborative study between the American and Dutch children oncology groups (COG and DCOG) included patients from three clinical COG/DCOG trials and also young adults less than 39 years of age in the Therapeutically Applicable Research to Generate Effective Treatments (TARGET) AML initiative [45]. Analysis of the different cohorts revealed similarly unfavorable outcomes with an EFS of 14-25% and an OS of 15-40% for patients with* FLT3-ITD *and* WT1 *mutations and/or the* NUP98-NSD1 *translocation [45]. In contrast to our findings however, the authors reported an EFS range of 15-35% in patients with* FLT3-ITD *only, which is lower than that achieved with current protocols, for which an EFS of 45% and an OS of 73% were found for patients with* FLT3-ITD* only. Notably, in the American-Dutch study, patients with co-occurrence of* NPM1 *mutations and* FLT3-ITD *(and without* WT1* and* NUP98-NSD1*) were separated from patients with* FLT3-ITD *only and had a slightly increased, albeit probably not statistically significant, survival. Similarly, we have previously observed favorable outcomes for patients with* NPM1 *mutations in their AML blasts with normal karyotype and proved this impact was not affected by the presence of* FLT3-ITD* [46]. In the current cohort, five patients were positive for mutations in* FLT3-ITD* and* NPM1* and negative for* WT1* and* NUP98* alterations. At present, four patients with a normal karyotype are still in first CCR, and the fifth patient with a complex karyotype and an* FLT3-ITD* AR >11 experienced early death. In summary, the principle findings of this American-Dutch study and the present study are very similar. However, the treatment outcomes for our patient groups are superior, most likely due to the fact that we included only patients between 0 and 18 years of age treated in Germany according to two contemporary protocols from the AML BFM study group.

## 5. Conclusion

Despite the fact that our study was partly based on data collected prospectively since 2004 and partly on data assessed* de novo* on stored material by either NGS or PCR, we can safely conclude that co-occurrence of the three factors, mutated* WT1 *and* FLT3-ITD and/or NUP98-NSD1 *translocation, still defines a subgroup of AML patients with devastating EFS and OS outcome, even with our current treatment protocols. Although the number of pediatric AML patients available for analysis of these three risk factors was limited and therefore not all interesting factors could be assessed in multivariate analysis, it is obvious that patients with double or triple mutations benefitted very little from the improved EFS and OS in our AML-BFM studies in recent years. Thus, for these pediatric patients, new and more targeted approaches are urgently needed for both first- and second-line treatments.

## Figures and Tables

**Table 1:** Patient Characteristics.

FEATURES		All patients		*WT1 *wild-type		*WT1 *mutated			*FLT3-ITD *neg.		*FLT3-ITD *pos.		
		n	%	n	%	n	%	p*∗*	n	%	n	%	p*∗*
*Study Population *													

*Number (*%)		353	100	305	100	48	100		301	100	52	100	

*Age* (years), median (range)		9.09		7.97		10.68		0.03	7.8		12.95		0.0001
		(0 -18)		(0 - 18)		(0.8 - 17.8)			(0 -18)		(2.7 - 17.9)		

*Gender*													
male		183	52%	159	52%	24	50%	0.783	153	51%	30	58%	0.36
female		170	48%	146	48%	24	50%		148	49%	22	42%	

*WBC count at diagnosis*													

median x 10^9^ cells/L (range)		24.5		24		42.85		0.1	20.1		73.5		0.0001
		(0.019 - 475)		(0.23 - 475)		(0.019 - 324)			(0.019-475)		(1.8 - 324)		

*Morphological Classification*													

FAB	M0	9	3%	7	2%	2	4%		5	2%	4	8%	
	M1/M2	134	38%	107	35%	27	56%		100	33%	34	65%	
	M4Eo+	31	9%	27	9%	4	8%		29	10%	2	4%	
	M4Eo-/M5	132	37%	125	41%	7	15%	0.008	121	40%	11	21%	0.0001
	M6	3	1%	2	1%	1	2%		3	1%	0	0%	
	M7	22	6%	21	7%	1	2%		22	7%	0	0%	
	AUL/other	22	6%	16	5%	6	13%		21	7%	1	2%	

*Cytogenetics*													

	t(8;21)	38	11%	38	12%	4	8%		36	12%	2	4%	
	inv(16)	32	9%	28	9%	0	0%		31	10%	1	2%	
	MLL rearr.	61	17%	60	20%	1	2%	<0.0001	60	20%	1	2%	0.0001
	others	117	33%	99	32%	18	38%		97	32%	20	38%	
	normal	97	27%	72	24%	25	52%		69	23%	28	54%	
	no data	8	2%	8	3%	0	0%		8	3%	0	0%	

*NUP98-NSD1*													

	positive	15	4%	7	2%	8	17%	<0.0001	6	2%	9	17%	<0.0001
	negative	231	65%	198	65%	33	69%		197	65%	34	65%	
	no data	107	30%	100	33%	7	15%		98	33%	9	17%	

*Co-mutations*													

*FLT3-ITD*	negative	301	85%	272	89%	29	60%	<0.0001					
	positive	52	15%	33	11%	19	40%						

*WT1*	wildtype	305	86%						272	90%	33	63%	0.0001
	mutated	48	14%						29	10%	19	37%	

*NPM1*	wildtype	316	90%	273	90%	43	90%		277	92%	39	75%	
	mutated	31	9%	26	9%	5	10%	0.69	20	7%	11	21%	0.0004
	no data	6	2%	6	2%	0	0%		4	1%	2	4%	

*nRAS*	wildtype	282	80%	245	80%	37	77%		235	78%	47	90%	
	mutated	59	17%	48	16%	11	23%	0.26	57	19%	2	4%	0.008
	no data	12	3%	12	4%	0	0%		9	3%	3	6%	

*KIT*	wildtype	308	87%	267	88%	41	85%	0.51	266	88%	42	81%	0.31
	mutated	41	12%	34	11%	7	15%		33	11%	8	15%	
	no data	4	1%	4	1%	0	0%		2	1%	2	4%	

*CEBPA*	wildtype	163	46%	121	40%	42	88%	<0.0001	122	41%	41	79%	<0.0001
	single	6	2%	4	1%	2	4%		6	2%	0	0%	
	double	10	3%	9	3%	1	2%		8	3%	2	4%	
	no data	174	49%	174	57%	0	0%		165	55%	9	17%	

*HSCT*													

HSCT in 1ts CR		64	18%	56	18%	8	17%	0.78	53	18%	11	21%	0.54
Chemotherapy only		289	82%	249	82%	40	83%		248	82%	41	79%	

*Patient Status*													

	alive	251	71%	221	72%	30	63%		225	75%	26	50%	
	deceased	73	21%	58	19%	15	31%	0.14	51	17%	22	42%	0.0001
	LFU	29	8%	26	9%	3	6%		25	8%	4	8%	

n, number; *WT1*, *Wilms tumor 1*; *FLT3-ITD, fms related tyrosine kinase 3*-internal tandem duplication; WBC, white blood cell; FAB, French-American-British; M4Eo+, AML M4 subtype with the presence of atypical eosinophils; M4Eo-, AML M4 subtype without the presence of atypical eosinophils; AUL, acute undifferentiated leukemia; t, translocation; inv, inversion; MLL rear., rearrangement of MLL gene; *NUP98-NSD1*, *Nucleoporin-Nuclear Receptor Binding SET Domain Protein 1* fusion gene; *NPM1*, *nucleophosmin 1*; *NRAS, neuroblastoma RAS viral oncogene homolog; c-KIT*, *KIT* proto-oncogene; *CEBPA*, *CCAAT/enhancer binding protein (C/EBP) alpha*; HSCT, hematopoietic stem cell transplantation; CR, complete remission; HSCT, hematopoietic stem cell transplantation; LFU, lost to follow-up. *∗*p-values derived from Pearson's Chi-squares test.

**Table 2:** Characteristics of *WT1* Variants.

UPN	exon	seq. read	mutation sequence^a^	amino acid alteration	VF (%)	dbSNP or COSMIC ID	published	previously reported sample	outcome
*missense substitutions*									

8	9		c.1333C>T	p.Arg445Trp	19.1	rs121907900, COSM21417	Yes	WT	CCR

15	9		c.1345C>A	p.Leu449Met	5.49		No		CCR

20	9		c.1385G>A	p.Arg462Gln	47.21	rs121907903, COSM4191067	Yes	AML, colon cancer, adenocarcinoma	CCR

21	9		c.1343A>G	p.His448Arg	33.12	COSM7335365	Yes	AML, mesothelioma	CCR

23	9		c.1333C>T	p.Arg445Trp	72.42	rs121907900	Yes	WT, DDS	CCR

26	7		c.1097C>G	p.Ser366Cys	2.57		No		CCR

35	9	different	c.1334G>A	p.Arg445Gln	3.12	rs121907903, COSM4191067	Yes	AML, colon cancer, adenocarcinoma	Relapse
	9	different	c.1307G>A	p.Cys436Tyr	44.21	COSM21438	Yes	AML	

*nonsense substitutions/insertions, deletions or duplications*									

1	7		c.1090_1093dupTC	p.Ala365Valfs*∗*4	43	COSM5487332	Yes	AML	CCR

2	7		c.1048-4_1056dupGCAGGATGTGCGA	p.Arg353Alafs*∗*19	30.25		No		LFU in CCR

3	7		c.1087_1161dup74	p.Lys387Asnfs*∗*44	n.d.		No		Relapse

4	7	different	c.1087_1091dupCGGTC	p.Ala365Glyfs*∗*69	5.08	COSM28954	Yes	AML, T-ALL	Relapse
4	7	different	c.1091C>A	p.Ser364*∗*	28.38	COSM27307	Yes	AML, WT	

5	7		c.1083_1098delTGTACGGTCGGCATCT	p.Val362Argfs*∗*65	46.82		No		NR/PR

6	7		c.1059dupT	p.Val354Cysfs*∗*14	35.9	COSM1317324	Yes	AML	Relapse

7	7		c.1179dupG	p.His394Alafs*∗*8	25		No		CCR

9	7	different	c.1078_1079insGCCGA	p.Thr360Serfs*∗*74	38.7		No		NR/PR
	7	different	c.1084_1085insGC	p.Val362Glyfs*∗*71	52.9		No		

10	7		c.1074_1077dupCCCG	p.Thr360Profs*∗*9	9.9		No		CCR

11	7		c.1079_1090delCTCTTGTACGGTinsTGGG	p.Thr360Metfs*∗*5	55.23		No		CCR

12	7		c.1058_1059insGA	p.Val354Metfs*∗*5	31.6		No		CCR

13	7	different	c.1058_1059insGGTG	p.Pro355Cysfs*∗*14	5.6		No		Relapse
	7	different	c.1078_1084dupACTCTTG	p.Val362Aspfs*∗*8	8.3	COSM5879281	Yes	AML	

14	7		c.1090_1093dupTCGG	p.Ala365Valfs*∗*4	22.81	COSM21392	Yes	AML	CCR

16	7		c.1054_1084dup	p.Val362Alafs*∗*16	7.3		No		CCR

17	7		c.1087delCinsGGG	p.Arg363Glyfs*∗*70	24.3		No		CCR

18	7		c.1054_1055insT	p.Arg352Leufs*∗*16	67.2	COSM5751511	Yes	T-ALL	CCR

19	7		c.1077_1078insTGTTTCTTCCGCCCAG	p.Thr360Cysfs*∗*13	36.95		No		Relapse

22	7		c.1087delCinsGG	p.Arg363Glyfs*∗*5	41.88		Yes	AML	CCR

24	7		c.1083_1090dupTGTACGGT	p.Ser364Leufs*∗*71	3.8	COSM27309	Yes	AML	CCR

25	9	different	c.1323_1338dupAAAGTTCTCCCGGTCC	p.Asp447Lysfs*∗*18	40.1		No		CCR
	9	different	c.1322_1332dupGAAAGTTCTCC	p.Arg445Glufs*∗*9	40.5		No		

27	7	different	c.1077_1078insGTTG	p.Thr360Valfs*∗*9	43.71		No		CCR
	7	different	c.1089dupG	p.Ser364Valfs*∗*4	49.24	COSM28966	Yes	AML	

28	7	different	c.1058delGinsCCA	p.Arg353Profs*∗*6	19.72		No		NR/PR
	7	different	c.1054_1055insAAAAAGATT	p.Arg352delins4	19.55		No		

29	7		c.1179dupG	p.His394Alafs*∗*8	25		No		CCR

30	7		c.1048_1057delGATGTGCGACinsAAGG	p.Asp350_Arg353	46.34		No		CCR

31	7		c.1093dupG	p.Ala365Glyfs*∗*3	44.49		Yes	AML	NR/PR

32	7		c.1048-8_1055dupGCCTGCAGGATGTGCG	p.Arg353Profs*∗*20	2.5		No		NR/PR

33	7		c.1090_1091dupTC	p.Ala365Argfs*∗*68	44.25	COSM28955	Yes	AML	Relapse

34	7	different	c.1087delCinsGA	p.Arg363Glufs*∗*5	4.1		No		NR/PR
	7	different	c.1086dupA	p.Arg363Thrfs*∗*5	5.33	COSM1166631	Yes	AML	
	7	different	c.1090_1093dupTC	p.Ala365Valfs*∗*4	36.29	COSM5487332	Yes	AML	

36	7	different	c.1090_1093dupTCGG	p.Ala365Valfs*∗*4	6.94	COSM5487332	Yes	AML	NR/PR
	7	different	c.1091dupC	p.Ala365Glyfs*∗*3	39.42	COSM27304	Yes	AML	

37	7	different	c.1057delCinsGG	p.Arg353Glyfs*∗*15	42.78		Yes	AML	CCR
	7	different	c.1087delCinsGGG	p.Arg363Glyfs*∗*70	52.11		No		

38	7		c.1068_1076delAGTAGCCCCinsGACGGTCGTTATTA	p.Val357Thrfs*∗*77	42.14		No		CCR

39	7		c.1087delCinsGG	p.Arg363Glyfs*∗*5	47.54		Yes	AML	CCR

40	7	different	c.1058_1059insGGTGCCGCTCG	p.Gly356Leufs*∗*6	48.49		No		Early Death
	7	different	c.1082_1091dupTTGTACGGTC	p.Ala365Cysfs*∗*6	41.83	COSM27303	Yes	AML	

41	7	different	c.1123dupA	p.Met375Asnfs*∗*9	44.5		Yes	AML	CCR
	7	different	c.1057_1058insTA	p.Arg353Leufs*∗*6	45.8		No		

42	7		c.1051_1055dupGTGCG	p.Arg353Cysfs*∗*7	34.73		No		Relapse

43	7		c.1058delGinsCC	p.Arg353Profs*∗*15	44.78	COSM28946	Yes	AML	Relapse

44	7	different	c.1079_1101delinsGAA	p.Thr360Argfs*∗*4	20.37		No		Relapse
	7	different	c.1088_1089insCTCGG	p.Ala365Glyfs*∗*69	10.69		No		

45	7		c.1090_1091insAGGT	p.Ser364*∗*fs*∗*1	42.97		No		Relapse

46	7		c.1058delGinsCC	p.Arg353Profs*∗*15	51.08	COSM28946	Yes	AML, T-ALL	NR/PR

47	7	different	c.1048-3_1055dupCAGGATGTGCG	p.Val354Metfs*∗*8	2.49		No		CCR
	7	different	c.1053dupG	p.Arg352Alafs*∗*16	3.83	COSM28980	Yes	AML	
	7	different	c.1054delCinsGG	p.Arg352Glyfs*∗*16	35.86	COSM28970	Yes	AML, T-ALL	

48	7		c.1089_1090insGGCCTCTTGTACGG	p.Ser364Glyfs*∗*73	40.49		No		Relapse

UPN, unique patient number; Seq. read, sequence read; VF, variant allele frequency; dup, duplication; ins, insertion; indel, Insertion-deletion; fs, frame-shift; *∗*termination codon; WT, Wilms tumor; DDS, Denis-Drash syndrome; T-ALL, T-cell acute lymphoblastic leukemia; CCR, continued complete remission, NR, non-response; PR, partial response; LFU, lost to follow-up.

^a^Transcript ID: NM_000378 was used to describe all alterations.

**Table 3:** Multivariate analysis.

*Cox regression analysis - Event-free survival*				

*Parameters*	Hazard ratio	95% confidence interval		p value
		Lower limit	Upper limit	

*WT1* mutation	0.79	0.41	1.53	0.479

*FLT3-ITD* AR ≥ 0.4	1.55	0.69	3.51	0.288

*WT1 *mutation, *FLT3-ITD* ≥ 0.4 and	3.88	1.42	10.66	0.008
*NUP98-NSD1* interaction				

t(8;21) and/or inv(16)	0.51	0.27	0.96	0.037

Unsatisfactory early response to treatment^a^	1.31	0.79	2.18	0.294

HSCT^b^	0.25	0.1	0.64	0.004

*Cox regression analysis - Overall survival *				

*WT1* mutation	0.84	0.35	2.06	0.710

*FLT3-ITD* ≥ 0.4	1.51	0.57	4.02	0.404

*WT1 *mutation, *FLT3-ITD ≥ 0.4 *and	3.42	1.04	11.21	0.042
*NUP98-NSD1* interaction				

t(8;21) and/or inv(16)	0.45	0.16	1.31	0.143

Unsatisfactory early response to treatment^a^	1.21	0.61	2.42	0.589

HSCT^b^	1.18	0.51	2.73	0.700

*WT1*, *Wilms tumor 1*; *FLT3-ITD*, *fms related tyrosine kinase 3-*internal tandem duplication; *NUP98-NSD1*, *Nucleoporin-Nuclear Receptor Binding SET Domain Protein 1* fusion gen; t, translocation; inv, inversion; HSCT, hematopoietic stem cell transplantation.

^a^Unsatisfactory early response to treatment was defined as persistence of >5% blasts in bone marrow at day 15 and/or 28 after treatment. ^b^hematopoietic stem cell transplantation events at first complete remission or after no-response to other treatments were included in the multivariate analysis as a time-dependent variable.

## Data Availability

The data used to support the findings of this study are included within the article.
